# The relationship between cognitive test anxiety and mindfulness among university students

**DOI:** 10.1192/j.eurpsy.2021.1634

**Published:** 2021-08-13

**Authors:** P. Ünal-Aydın, M. Sevinç, Y. Arslan, M. Güçlü, O. Aydın

**Affiliations:** Psychology, International University of Sarajevo, Faculty of Arts and Social Sciences, Sarajevo, Bosnia and Herzegovina

**Keywords:** Cognitive, stress, test anxiety, mindfulness

## Abstract

**Introduction:**

Cognitive test anxiety is acknowledged as intense anxiety that prevents the effective use of the previously learned knowledge during the exam and leads to a decrease in success. Mindfulness is indicated as the ability to bring one’s attention to experiences in the present moment in a non-judgmental way. Despite promising outcomes of mindfulness techniques in regulating stress levels, much uncertainty still exists about the specific associations between cognitive test anxiety and mindfulness subcategories.

**Objectives:**

The aim of this study was to investigate the relationship between cognitive test anxiety and subcategories of mindfulness among university students which may help improving current mindfulness interventions that show promising results to tackle cognitive test anxiety.

**Methods:**

One hundred-eighty-two university students were recruited for the study via online forms. Mindfulness was measured with Five Facet Mindfulness (FFMQ-S) and the cognitive test anxiety was assessed with Cognitive Test Anxiety Scale-Revised (CTAR).

**Results:**

Total scores of CTAR-R has an association between subscales of FFMQ; act-aware and non-judge in a positive direction, whereas; observe and describe in a negative direction. In addition, according to our regression model, FFMQ subscales of describing to indicated lower levels of CTAR scores, whereas act aware and non-judge indicated higher levels of CTAR scores.

**Conclusions:**

The findings of the study partially corroborated the previous results by offering inferences about the subcategories of mindfulness. Additionally, these findings suggest that current interventions may target specific subcategories of mindfulness to maximize the positive outcomes of the treatment.

**Disclosure:**

No significant relationships.

